# Risk Factors for Pulmonary Complications After Major Oral and Maxillofacial Surgery With Free Flap Reconstruction

**DOI:** 10.7759/cureus.50408

**Published:** 2023-12-12

**Authors:** Yukie NItta, Masanori Iwasaki, Kanta Kido

**Affiliations:** 1 Division of Dental Anesthesiology, Department of Oral Pathological Science, Faculty of Dental Medicine and Graduate School of Dental Medicine, Hokkaido University, Sapporo, JPN; 2 Division of Preventive Dentistry, Department of Oral Health Science, Graduate School of Dental Medicine, Hokkaido University, Sapporo, JPN

**Keywords:** risk factors, anesthesia time, free flap reconstruction, oral and maxillofacial surgery, postoperative pulmonary complications

## Abstract

Background

Postoperative pulmonary complications (PPCs) are common and result in increased morbidity and mortality. A variable incidence of PPCs has been reported in patients who have undergone major oral and maxillofacial surgery with free flap reconstruction, which is one of the most extensive forms of head and neck cancer surgery, and perioperative risk factors for PPCs in these patients have not been fully elucidated. Furthermore, the ARISCAT (Assess Respiratory Risk in Surgical Patients in Catalonia) score and Gupta risk index have not been investigated in patients undergoing head and neck cancer surgery. This study aimed to identify perioperative risk factors for PPCs after major oral and maxillofacial surgery with free flap reconstruction.

Methodology

This was a single-center, retrospective cohort study of 118 patients who had undergone major oral and maxillofacial surgery with free flap reconstruction between 2009 and 2020. PPCs were defined as pneumonia, hypoxemia caused by atelectasis, pleural effusion, pulmonary embolism, pulmonary edema, bronchospasm, pneumothorax, and acute respiratory failure. Predictors of PPCs were identified in univariate and multiple Poisson regression analyses.

Results

The incidence of PPCs was 18.6% (22/118 patients). The most frequent PPC was pneumonia. No preoperative patient-related parameter was identified to predict PPCs. In univariate analysis, the only predictor was anesthesia time ≥1,140 minutes (odds ratio = 3.0, p = 0.036). Multivariable Poisson regression identified two independent predictors of PPCs, namely, anesthesia time ≥1,140 minutes (incidence rate ratio (IRR) = 2.18, 95% confidence interval (CI) = 1.1-4.3, p = 0.024) and a large amount of intraoperative fluid (IRR = 1.00018, 95% CI = 1.000018-1.000587, p = 0.037).

Conclusions

Patients undergoing major oral and maxillofacial surgery with free flap reconstruction are at high risk of PPCs. Longer anesthesia time and administering a large amount of fluid during surgery were significantly correlated with the risk of PPCs.

## Introduction

Postoperative pulmonary complications (PPCs) are common and associated with unfavorable outcomes, including prolonged hospital stay and increased morbidity and mortality [1,2]. The prevalence of PPCs is estimated to be 1%-23% and varies depending on the patient’s general condition and surgical factors [3]. Many risk factors contribute to PPCs, including age, chronic obstructive pulmonary disease, smoking, American Society of Anesthesiologists (ASA) classification, functional impairment, low serum albumin (<3.5 mg/dL), preoperative oxygen saturation, history of respiratory infection, duration of surgery of more than three hours, emergent surgery, and surgical site (intrathoracic, intraabdominal, head and neck, and neurosurgical) [4,5].

Head and neck surgery, in particular, has been identified as a risk factor for the development of PPCs [6]. Previous studies have reported a variable incidence of PPCs among patients undergoing head and neck cancer surgery (HNCS), especially major oral surgery with free flap reconstruction, which is one of the most extensive forms of HNCS. The reported incidence rate for PPCs after HNCS ranges from 1.9% to 44.8% [7,8]. However, information on risk factors for PPCs after major oral and maxillofacial oral surgery with free flap reconstruction is scarce, especially concerning intraoperative indices, such as anesthesia time [9]. For example, the ARISCAT (Assess Respiratory Risk in Surgical Patients in Catalonia) score [2] and Gupta risk index [10] were developed using large surgical populations but included few head and neck surgery cases. Furthermore, no distinction is made between major HNCS and minor surgery [11]. Therefore, the performance of existing models for the prediction of PPCs in patients undergoing HNCS is likely to be poor.

This single-center, retrospective, cohort study aimed to identify perioperative risk factors for PPCs after major oral and maxillofacial surgery with free flap reconstruction.

## Materials and methods

This study was approved by the Institutional Review Board of Hokkaido University Hospital (clinical study code: 021-0037) and was conducted in accordance with the principles of the Declaration of Helsinki and its amendments. The need for informed consent was waived by the Institutional Review Board because broad consent was obtained before general anesthesia.

Study population

A total of 118 patients who underwent major oral and maxillofacial surgery with free flap reconstruction in the Department of Oral Diagnosis and Medicine or Department of Oral and Maxillofacial Surgery at Hokkaido University Hospital during the 12-year period between January 1, 2009, and December 31, 2020, were systematically reviewed. The study exclusion criteria were incomplete medical records, reoperation within five days, maintenance with total intravenous anesthesia, and no free tissue transfer reconstruction.

Surgical procedure

Tracheotomy was performed under general anesthesia at the start of surgery and was followed by major tissue resection in all cases. The defect was reconstructed with an appropriate microvascular free flap under a microscope by a plastic surgeon. The flap was harvested from the donor site and inserted into the wound, after which the operation was completed.

Intraoperative and postoperative management

All patients were anesthetized using an inhalational anesthetic (sevoflurane or desflurane) with narcotics (fentanyl and remifentanil) and a muscle relaxant (rocuronium). Patients were intubated orally or nasally before tracheostomy. A gastric feeding tube was inserted before the surgery in all cases. Cephem antibiotics were used if the patient did not have an allergy to them. Preoperative antibiotics were administered at the time of anesthesia induction as ordered by the oral surgeon. During anesthesia, antibiotics were administered every few hours. Antibiotics were also administered postoperatively in the same way. During the operation, we continuously measured the body temperature of all patients and warmed them using forced air warming. After the operation, intensive care unit (ICU) doctors and ward doctors continuously measured the patient’s body temperature and warmed the patient as necessary to avoid hypothermia. After completion of surgery, the patients were returned to the ward if breathing spontaneously or to the ICU if in an anesthetized state. In the ICU, patients were sedated and ventilated at the discretion of the intensivist. Sedation was reduced in the ICU if the patient was stable on postoperative day one. The patient was then weaned from ventilatory support and discharged from the ICU back to the ward. The patients who were maintained in the ICU were sedated and under invasive mechanical ventilation for about 12 hours. All patients were kept on bedrest for 72 hours postoperatively for flap healing. For the prevention of deep vein thrombosis, mechanical methods of prophylaxis (such as graduated compression stockings and intermittent pneumatic compression devices) were used perioperatively.

Data collection

All patient data were collected from preoperative examination notes, medical charts, and anesthetic records. Preoperative data included sex, age, body mass index (BMI), comorbidities (hypertension, ischemic heart disease, respiratory dysfunction, diabetes, cerebrovascular disease, liver disease, and renal insufficiency), and smoking history. Patients were considered to be smokers if they were smoking currently or had a smoking history. ASA physical status was recorded. Preoperative laboratory results were recorded, including serum albumin and hemoglobin, as were findings on respiratory function tests. Intraoperative data included operation time, anesthesia time, intraoperative administration of fluids, blood loss, and transfusion.

Diagnosis of PPCs

Referring to previous studies [8,12], PPCs were defined as pneumonia, hypoxemia caused by atelectasis, pleural effusion, pulmonary embolism, pulmonary edema, bronchospasm, pneumothorax, and acute respiratory failure. Pneumonia included treatment with antibiotics for suspected respiratory tract infection. PPCs were diagnosed by a respiratory physician or attending oral surgeon and identified in critical care reports.

Statistical analysis

The patients were divided into two groups depending on the presence or absence of PPCs during the five days after surgery. Categorical data were compared between the two groups using Fisher’s exact test. Data with a nonparametric distribution are summarized as the median (interquartile range (IQR)) and were compared between groups using the Mann-Whitney U test. In the event of a high incidence of outcomes, logistic regression might have overestimated the risk of association. Therefore, multivariable Poisson regression with a robust variance estimator was performed to investigate the association between PPCs and some perioperative parameters [13]. The results are presented as the incidence rate ratio (IRR) and 95% confidence interval (CI). All statistical analyses were performed using EZR version 1.52 (Saitama Medical Center, Jichi Medical University, Saitama, Japan) and Stata version 18 (StataCorp, College Station, TX, USA). All statistical tests were two-sided, and a p-value less than 0.05 was considered statistically significant.

## Results

Patient characteristics

A total of 137 patients met the inclusion criteria and their charts were systematically reviewed. In total, 19 patients were excluded because of incomplete medical records (n = 6), reoperation within five days (n = 11), maintenance with total intravenous anesthesia (n = 1), or no free tissue transfer (n = 1). A total of 118 patients were included in the analysis.

Table 1 shows the patient characteristics and perioperative data. Most patients (n = 102, 86.4%) were diagnosed with oral cancer; the remaining non-malignant cases included ameloblastoma and osteoradionecrosis (data not shown). The median age was 65 years (IQR = 58, 71), the median BMI was 21.1 (IQR = 18.7, 22.7), and 66.1% of patients had a history of smoking.

**Table 1:** Univariate analysis: comparison of characteristics between the postoperative pulmonary complications (PPCs) group and non-PPCs group. *: Smoking histories: current smokers or patients with smoking history. IQR = interquartile range; BMI = body mass index; VC = vital capacity; %VC = % vital capacity; FEV1.0% = forced expiratory volume % in one first) second; FEV1.0 = forced expiratory volume in one second; ASA = American Society of Anesthesiologists; ICU = intensive care unit

Variables	Total (N = 118)	Non-PPC group (96)	PPC group (22)	Odds ratio	P-value
Demographic					
Sex, male, N (%)	73 (62)	57 (59.4)	16 ( 72.7)	1.82	0.332
Median, age (IQR), years	65 (58–71)	65 (57–71)	64 (60–74)		0.52
Median, BMI (IQR), kg/m^2^	21.1 (18.7–22.7)	21.41 (18.5–23.21)	20.89 (19.51–22.31)		0.89
*Smoking history, N (%)	78 (66.1)	64 (66.7)	14 ( 63.6)	0.88	0.806
Median VC (IQR), L	3.33 (2.88–4.07)	3.28 (2.89–4.06)	3.66 (3.08–4.51)		0.269
Median %VC (IQR)	106.0 (95.78–117.93)	106.00 (95–117.78)	104.00 (98–117.98)		0.917
Median FEV1.0 (IQR), L	2.48 (2.01–3.10)	2.48 (2.02–3.09)	2.44 (1.99–3.43)		0.99
Median FEV1.0% (IQR)	75.9 (69.78–80.00)	75.70 (70.23–81.00)	76.80 (68.42–79.76)		0.79
Median serum albumin (IQR), g/dL	4.0 (3.7–4.2)	4.00 (3.7–4.2)	4.05 (3.7–4.3)		0.53
Median hemoglobin (IQR), mg/dL	12.9 (11.5–14.0)	12.90 (11.50–14.05)	12.60 (11.63–13.50)		0.63
ASA2, N (%)	92 (78)	72 (75)	20 (90.9)	3.31	0.154
Hypertension, N (%)	42 (35.6)	33 (34.4)	9 (40.9)	1.32	0.625
Ischemic heart disease, N (%)	4 (3.4)	4 (4.2)	0 (0.0)	0	1
Respiratory dysfunction, N (%)	51 (43)	39 (40.6)	12 (54.5)	1.75	0.245
Diabetes mellitus, N (%)	25 (21.2)	18 (18.8)	7 (31.8)	2.01	0.245
Cerebrovascular disease, N (%)	11 (9)	8 ( 8.3)	3 (13.6)	1.73	0.428
Liver disease, N (%)	16 (13.5)	11 (11.5)	5 (22.7)	2.25	0.176
Renal insufficiency, N (%)	23 (19.5)	17 (17.7)	6 (27.3)	1.73	0.371
Perioperative					
Median operative time (IQR), minutes	905 (809–1,019)	887 (806–1,001)	968 (857–1,083)		0.126
Median anesthesia time (IQR), minutes	988 (888–1,098)	973 (886–1,088)	1,036 (973–1,145)		0.069
Anesthesia time ≥1,140 minutes, N (%)	23 (19.5)	15 (15.6)	8 (36.4)	3.1	0.037
Median intraoperative fluids (IQR), mL	5,100 (4,275–5,950)	5,055 (4,200–5,813)	5,955.00 (4,587.5–6,687.5)		0.082
Median blood loss (IQR), mL	593 (325–934)	535.00 (317.50–930.00)	697.50 (376.25–1,106.25)		0.185
Intraoperative transfusion ≥4 units, N (%)	70 (59.3)	55 (57.3)	15 ( 68.2)	1.59	0.471
Postoperative					
Postoperative care in an ICU, N (%)	72 (61)	60 (83.3)	12 (16.7)	0.72	0.629

Table 1 summarizes the preoperative variables (findings on preoperative examination and medical comorbidities) and outcomes. The patients were categorized mainly as ASA class 2 (78%), followed by ASA class 1 (22%). The most prevalent medical comorbidities were respiratory dysfunction (43%), hypertension (35.6%), and diabetes mellitus (21%). The median operation time was 905 minutes (IQR = 809, 1019). The median anesthesia time was 988 minutes (IQR = 888, 1098). In total, 72 (64.4%) patients were transferred to the ICU and maintained under anesthesia and mechanical ventilation via tracheostomy.

Incidence of PPCs

Table 2 shows the incidence of the individual PPCs. Of the 118 patients, 22 (18.6%) developed one or more PPCs. One patient developed two pulmonary complications. Overall, 19 (82.6%) patients developed pneumonia and two developed hypoxemia caused by atelectasis (8.8%). None of the patients developed pleural effusion, pulmonary embolism, pulmonary edema, or respiratory failure.

**Table 2:** Frequency of individual postoperative pulmonary complications (PPCs). Number of patients who developed PPCs was 22. One patient sustained more than one pulmonary complication.  None of the patients developed pleural effusions, pulmonary embolism, pulmonary edema, or respiratory failure.

Pulmonary complication	Number of incidences	Proportion of total PPCs observed (%)
Pneumonia or suspicion of pneumonia	19	82.6
Hypoxemia caused by atelectasis	2	8.8
Bronchospasm	1	4.3
Pneumothorax	1	4.3
Total	23	100

Univariate and multivariate analyses of patient-related parameters

Table 1 shows the results of univariate analysis of the patient-related parameters. Regarding preoperative variables, there was no significant difference in the incidence of respiratory dysfunction or smoking history. PPCs developed in 21.7% of patients with ASA 2 but in only 7.7% with ASA 1. Analysis of intraoperative variables showed that patients who received a large amount of intraoperative fluids had a slightly increased risk of PPCs (p = 0.08). There was no significant difference in the amount of blood transfused intraoperatively between the two groups.

The patients were then divided into two groups and compared according to whether they were or were not in the top 20% for anesthesia time (≥1,140 minutes and <1,140 minutes, respectively). PPCs developed in 36.4% of the top 20% of cases but in only 15.6% of the cases that were not in the top 20%. The risk of PPCs was significantly higher in patients who were in the top 20% (odds ratio = 3.0, p = 0.036).

PPCs developed in 16.7% of patients who were transferred to the ICU in an anesthetized state but in 21.7% of those who were returned to the ward with spontaneous breathing. The difference was not statistically significant.

Table 3 shows the results of multivariate analysis of patient-related parameters. Independent perioperative predictors of PPCs were sought in a multivariable Poisson regression model. In addition to sex and age, the variables with a p-value of 0.2 in univariate analysis that were included in the multivariable model were anesthesia time, ASA physical status, and amount of intraoperative fluid administered. Anesthesia time ≥1,140 minutes (IRR = 2.18, 95% CI = 1.1-4.3, p = 0.024) and a large amount of intraoperative fluid (IRR = 1.00018, 95% CI = 1.000018-1.000587, p = 0.037) were found to be statistically significant.

**Table 3:** Multivariable Poisson regression analysis of postoperative pulmonary complications (PPCs). ASA = American Society of Anesthesiologists; CI = confidence interval; IRR = incidence rate ratio

Factor	IRR	95% CI (lower, upper)	P-value
Anesthesia time ≥1,140 minutes	2.175755	(1.1–4.3)	0.024
Age (year)	1.037633	(1.0–1.1)	0.088
ASA	2.827915	(0.65–12.31)	0.166
Sex, male	1.570599	(0.72–3.44)	0.258
Intraoperative fluids (mL)	1.000302	(1.000018–1.000587)	0.037

## Discussion

In this study, the incidence of PPCs after oral and maxillofacial surgery with free flap reconstruction was 18.6%. Although some patients with PPCs were treated by a respiratory specialist, none had severe respiratory symptoms or needed longer mechanical ventilation support or a longer ICU stay. None of the preoperative parameters identified to be risk factors in previous studies was associated with PPCs. Only prolonged anesthesia and a large amount of intraoperative fluid were identified to be perioperative risk factors in this study.

In addition to being a highly complex procedure and requiring prolonged surgical and anesthesia times, major oral surgery with free flap reconstruction is typically performed in patients who are elderly and have comorbidities [14]. Therefore, this type of surgery is associated with a high risk of PPCs developing. Reports on the incidence of PPCs in patients undergoing major oral surgery with free flap reconstruction vary, likely because of the use of different definitions of PPCs. For example, Logan et al. reported a 1.8% incidence of PPCs, which they defined as adult respiratory distress syndrome [7]. Meanwhile, Damian et al. reported a 32.7% incidence of PPCs, which they defined as pulmonary edema, pneumonia, pneumothorax, pulmonary embolism, or adult respiratory distress syndrome [12]. PPC incidence rates of 13.1%, 18.8%, and 15% have also been reported after major oral surgery with free flap reconstruction [1,4,15]. Therefore, the present study provides further evidence that this surgery is associated with a high risk of PPCs.

Patients at high risk of PPCs need earlier therapeutic intervention preoperatively, aggressive intraoperative management, and comprehensive postoperative management [16]. The ARISCAT risk score [2] and Gupta index [10] have been developed as decision support tools for use in patients at highest risk of PPCs, but these tools were developed mainly for use in general surgery populations and are unlikely to provide a correct estimate of the risk of PPCs after major head and neck surgery [11]. Indeed, these tools did not perform well in our study because both tools could not detect the risk of PPCs in our cases.

Previous studies have explored various preoperative risk factors for PPCs in patients undergoing major oral surgery with free flap reconstruction. Risk factors associated with this type of surgery include advanced age, male sex, higher BMI, longer smoking history, low serum albumin, pulmonary comorbidity, alcohol abuse, and low preoperative metabolic equivalents [1,2,4,7,10,12,14-16]. Several studies have found that a generally poor physical condition (higher ASA grade) is a risk factor for PPCs [1,4,10,16]. In this study, we did not find a relationship between the incidence of PPCs and any particular preoperative risk factor. Furthermore, ASA was not identified as a risk factor for PPCs, possibly because of the small sample size and our study population potentially being more healthy than those in the previous reports [1,4].

Previous studies have suggested that intraoperative parameters, including duration of surgery or anesthesia, amount of intraoperative fluid, and blood transfusion during surgery, are involved in the development of PPCs. Operation time of more than three hours is mentioned as a predictor of PPCs in the American College of Physicians guideline [16]. Weber et al. identified that protracted anesthesia was a significant risk factor for PPCs [17], and Loeffelbein et al. suggested that PPCs were significantly more likely after operation time >500 minutes (>8.3 hours) [1]. In contrast, Shaw et al. [9] and Damian et al. [12] found no association between the duration of anesthesia or surgery and the incidence of PPCs. In our study, anesthesia time ≥1,140 minutes was identified as a predictor of PPCs. There are several possible reasons for the association of surgery or anesthesia time with PPCs. Prolonged operation time causes an increased postoperative stress response and immunosuppressive state. The incidence of pneumonia is increased by prolonged mechanical ventilation [18], and atelectasis is an inevitable consequence of prolonged anesthesia and mechanical ventilation [19]. Prolonged operation time also increases the risk of fluid overload [1]. Our findings suggest that a large amount of fluid administered and prolonged anesthesia are predictors of PPCs.

Regarding postoperative management, patients who undergo major oral surgery with free flap reconstruction need to be kept on bed rest to prevent thrombosis at the site of the vascular anastomosis. Given that prolonged mechanical ventilation is a potential risk factor for PPCs, patients who undergo ventilation in the ICU after surgery are predisposed to these complications. Allak et al. reported that the incidence of pneumonia was lower in patients who were weaned immediately postoperatively compared with those in whom mechanical ventilation was continued in the ICU [20]. In contrast, Steven et al. found that the respiratory outcomes of immediate postoperative weaning from ventilation were not inferior to those after routine ICU admission [8]. In the present study, there was no significant difference between patients who were admitted to the ICU and those who were returned to the ward, possibly because of the extremely long anesthesia time in both groups. Another reason may be that the ICU stay was shorter in this study than previously reported [12].

This study had several limitations. First, because of the single-center, retrospective design and a long duration of data correction, there may be some bias in data collection and incomplete notes in case records (some physical habilitation scoring systems such as METs and Performance Status Score). Because of the long duration of data correction, there might have been some changes in perioperative protocols. Second, a limitation of this study was the lack of a control group for the methodological reason of a retrospective and long-duration study. Third, the sample size was smaller compared to other studies. Fourth, there is a possibility of overestimation or underestimation of the actual incidence of PPCs because some diagnoses of PPCs relied on evaluation by the attending oral surgeon. Lastly, our trigger for transfusion was discretionary.

## Conclusions

Patients undergoing major oral and maxillofacial surgery with free flap reconstruction are at high risk of PPCs. Regarding preoperative variables, there was no significant difference in the incidence of respiratory dysfunction or smoking history. Compared to patients who were returned to the ward with spontaneous breathing, the risk of PPCs was not significantly higher in patients who were transferred to the ICU in an anesthetized state. Longer anesthesia time and administering a large amount of fluid during surgery were significantly correlated with the risk of PPCs.
